# Interleukin-6 is better than C-reactive protein for the prediction of infected pancreatic necrosis and mortality in patients with acute pancreatitis

**DOI:** 10.3389/fcimb.2022.933221

**Published:** 2022-11-18

**Authors:** Jiarong Li, Zhenping Chen, Lei Li, Tianming Lai, Hao Peng, Ling Gui, Wenhua He

**Affiliations:** Pancreatic Disease Centre, Department of Gastroenterology, The First Affiliated Hospital of Nanchang University, Nanchang, China

**Keywords:** C-reactive protein, interleukin 6, infected pancreatic necrosis, mortality, organ failure, severe acute pancreatitis

## Abstract

**Introduction:**

This study aimed to identify whether interleukin-6 (IL-6) is better than C-reactive protein (CRP) for the prediction of severe acute pancreatitis (SAP), infected pancreatic necrosis (IPN), and mortality.

**Methods:**

Sixty-seven patients with acute pancreatitis (AP) who were hospitalized within 48 h of onset and received serum CRP and IL-6 tests from September 2018 to September 2019 were included. Spearman’s correlation was performed to assess their associations with severity. The areas under the curve (AUCs) for the prediction of SAP, organ failure, pancreatic necrosis, IPN, and mortality were estimated using receiver operating characteristic curves.

**Result:**

Serum CRP and IL-6 levels were significantly positively correlated with the severity of AP (p < 0.05). The AUC for the prediction of SAP based on the CRP level was 0.78 (95% CI, 0.66–0.89) and that based on the IL-6 level was 0.69 (95% CI, 0.56–0.82). For the prediction of organ failure and pancreatic necrosis, CRP was more accurate than IL-6 (AUC 0.80 vs. 0.72 and 0.75 vs. 0.68, respectively). However, CRP was less accurate than IL-6 for predicting mortality and IPN (AUC 0.70 vs. 0.75 and 0.65 vs. 0.81, respectively). Systemic inflammatory response syndrome plus CRP was more accurate than systemic inflammatory response syndrome plus IL-6 (AUC 0.79 vs. 0.72) for the prediction of SAP.

**Conclusions:**

IL-6 was more accurate than CRP for predicting mortality and IPN in patients with AP.

## Introduction

Acute pancreatitis (AP) is a common gastrointestinal disease with an increasing morbidity rate (Tenner et al., 2013). According to the 2012 revised Atlanta classification, the severity of AP is defined as mild acute pancreatitis (MAP), moderately severe acute pancreatitis (MSAP), and severe acute pancreatitis (SAP). Approximately 20% of patients with AP have severe disease, and 36%–50% of those with SAP die (Banks et al., 2013). It is critical to identify patients at risk of severe disease to facilitate the implementation of early active interventions to improve the prognosis of patients (Whitcomb, 2006; Talukdar and Swaroop Vege, 2011; He et al., 2017). Several multifactor scoring systems predict the severity of AP, such as the Acute Physiology and Chronic Health Evaluation II, the Ranson, the Bedside Index of Severity in Acute Pancreatitis, and the Glasgow scoring systems (He et al., 2017); however, the limitations of these scoring systems are the inability to obtain a complete score until at least 48 h into the illness, the complexity of the scoring system, or the poor accuracy (Matull et al., 2006; He et al., 2017). Moreover, clinicians also use individual laboratory parameters to assist in the prediction of which patients with AP will develop severe disease. C-reactive protein (CRP) and interleukin-6 (IL-6) are laboratory markers most commonly used to predict disease severity and prognosis (Meher et al., 2015).

CRP is an acute-phase reactant. A CRP level of >150 mg/L within the first 72 h strongly correlates with the presence of pancreatic necrosis (PN), and it was recommended in some guidelines for the prediction of SAP (Banks and Freeman, 2006; Greenberg et al., 2016; Leppaniemi et al., 2019). Clinicians widely consider CRP to be the gold standard for disease severity assessment at 48 h after disease onset (Staubli et al., 2015). IL-6 is released by a wide range of cells in response to tissue injury, and it stimulates the synthesis of acute-phase proteins, including CRP, by hepatocytes *in vitro* and *in vivo (* Bhatia and Moochhala, 2004). Nieminen et al. (2014) found that IL-6 levels on admission have prognostic value for SAP, and when measured within 48 h of AP onset, an IL-6 level of ≥28.90 pg/ml was reported to be the best biomarker among those tested (IL-8, IL-10, and CRP) in a prospective cohort study on the prediction of SAP (Jain et al., 2018). Moreover, Jain et al. found that the additional consideration of IL-6 significantly improved the predictive value of systemic inflammatory response syndrome (SIRS) for the prediction of SAP (Jain et al., 2018). In view of the finding that IL-6 is highly accurate for the prediction of SAP, our pancreatic disease center routinely detected IL-6 in patients with AP admitted in the early stage and recorded it in the AP database since September 2018. We designed this cohort study based on this prospective database to compare the accuracy of IL-6 detected within 48 h of onset with that of CRP for the prediction of SAP, organ failure (OF), PN, infected pancreatic necrosis (IPN), and mortality.

## Methods

### Ethical approval

The construction of the AP database and the performance of this study was conducted according to the Declaration of Helsinki and was approved by the Clinical Ethics Committee of the First Affiliated Hospital of Nanchang University (Approval No. (2011)001). Informed consent was waived.

### Patients

This study retrospectively screened 1,280 AP cases admitted to the First Affiliated Hospital of Nanchang University from September 2018 to September 2019. We selected patients who were admitted to the hospital within 48 h of disease onset and had values for IL-6 and CRP. All of the patient data in this retrospective cohort study were collected from the AP database. Serum IL-6 and CRP were tested using enzyme-linked immunosorbent assays at the Inspection Center of the First Affiliated Hospital of Nanchang University. Briefly, coated microtiter plates with anti-human IL-6 antibody and CRP antibody, and detected by double-antibody sandwich ELISA.

### Classification of acute pancreatitis severity

In this study, we classified the severity of AP at the time of discharge based on the occurrence of OF (respiratory, cardiovascular, and renal), systemic complications, and local pancreatic complications during the period from onset to hospital discharge. SIRS scores were calculated daily in the 7 days after admission. Patients with AP were divided into groups with MAP, MSAP, and SAP according to the revised Atlanta classification (Banks et al., 2013). The above definitions are explained in **Table 1**.

**Table 1:** Definitions of endpoints.

Endpoint	Definition
Systemic inflammatory response syndrome (SIRS)	Presence of two or more criteria: * Heart rate, >90 beats/min * Core temperature, <36°C or >38°C * White blood count, <4,000 or >12,000/mm * Respirations, >20/min or pCO_2_ <32 mm Hg
Persistent SIRS	SIRS lasted more than 48 h
Respiratory failure	PaO_2_/FiO_2_ ≤ 300, or requirement for mechanical ventilation
Cardiovascular failure	Circulatory systolic blood pressure <90 mm Hg, despite adequate fluid resuscitation, or requirement for inotropic catecholamine support
Renal failure	Creatinine level > 177 μmol/L after rehydration or new need for hemofiltration or hemodialysis
Persistent organ failure	Organ failure persists for >48 h
Systemic complications	Exacerbation of preexisting comorbidity
Necrotising pancreatitis.	Inflammation associated with pancreatic parenchymal necrosis and/or peripancreatic necrosis
Local complications	Include acute peripancreatic fluid collection, pancreatic pseudocyst, acute necrotic collection, walled-off necrosis, gastric outlet dysfunction, splenic and portal vein thrombosis, and colonic necrosis
Mild acute pancreatitis	Absence of organ failure and absence of local or systemic complications
Moderately severe acute pancreatitis	Presence of transient organ failure or local or systemic complications
Severe acute pancreatitis	Persistent organ failure.
Mortality	Patients who died during hospitalization or within 30 days of discharge

### Statistics

Demographic and baseline characteristics were analyzed using descriptive statistics. Qualitative variables are described as numbers and percentages. Quantitative variables are described as the means ± standard deviations. Medians and interquartile ranges (IQRs) were reported if the distribution of the variable was not normal. Kruskal–Wallis tests were performed for nonnormally distributed variables. The predictive accuracy was evaluated using the area under the receiver operating characteristic (ROC) curve (AUC). The best cutoff value was selected according to the largest value of the Youden index. The diagnostic characteristics were assessed with the AUC, sensitivity, specificity, positive likelihood ratio (+LR), and negative likelihood ratio (−LR). When analyzing the combination of SIRS and serum IL-6 or CRP levels for the prediction of SAP, univariate analyses were performed with SAP as the dependent variable and predefined prognostic factors as independent variables, including SIRS and the serum IL-6 and CRP levels. Logistic regression analysis was used to assess the combinations, and then ROC curve analysis was performed to evaluate the diagnostic value of the combinations of SIRS with IL-6 and SIRS with CRP for the severity of AP. Epi Info 7 (Centers for Disease Control and Prevention, Atlanta, GA, USA) and Microsoft Excel^®^ 2013 (Microsoft, Inc., Redmond, WA, USA) were used to collect and process the data, which were then analyzed with IBM SPSS statistics version 25.0 (IBM Corp., Armonk, NY, USA).

## Results

### Patient characteristics

**Figure 1:**
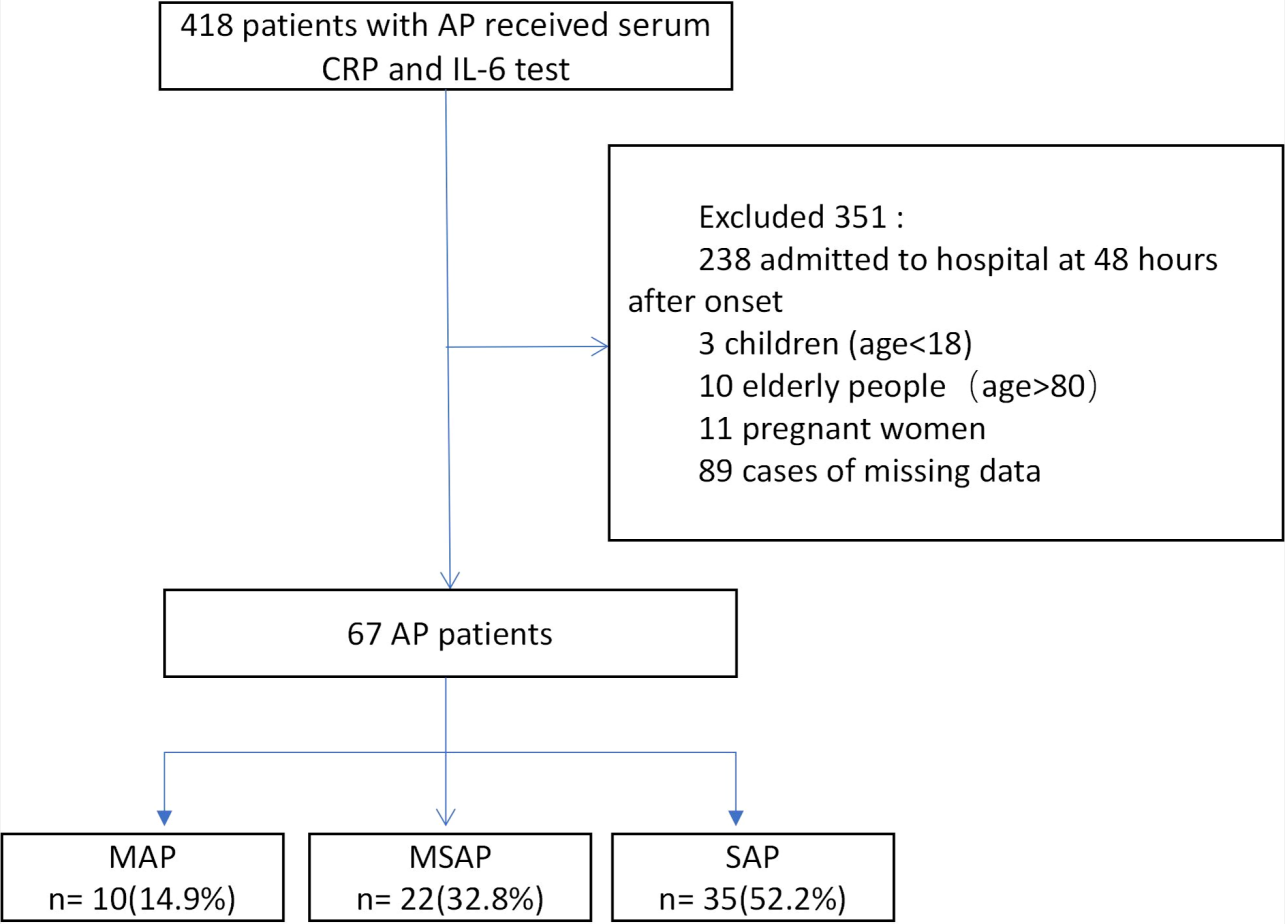
Study flowchart.

A total of 1,280 AP patients were admitted to the First Affiliated Hospital of Nanchang University from September 2018 to September 2019. Of these, 67 were eventually enrolled in this study after the application of the exclusion criteria (the details are listed in **Figure 1**). Their characteristics and the clinical outcomes of AP are shown in **Table 2**. The mean age of the included AP patients was nearly 48 years, and most patients were female. The causes of AP included hyperlipidemia (44.8%), biliary causes (37.3%), alcohol (14.9%), and idiopathic pancreatitis (3.0%). According to the 2012 Atlanta classification criteria, there were 35 (52.2%) patients with SAP, 22 (32.8%) patients with MSAP, and 10 (14.9%) patients with MAP. A total of 80.6% of the patients received treatment in the pancreatic intensive care unit, 46 (68.7%) patients developed OF, 34 patients developed persistent OF, and 9% died (**Table 2**).

**Table 2:** Patient demographics and baseline clinical.

Characteristic			*n* = 67
Age in years, mean (*SD*)			48 (17)
Sex, *n* (%)			
	Female		47 (70.1%)
	Male		20 (29.9%)
BMI			25 (23–28)
Cause of pancreatitis			
	Biliary		25 (37.3%)
	Hyperlipidemic		30 (44.8%)
	Alcoholic		10 (14.9%)
	Idiopathic		2 (3.0%)
Coexisting condition			
	Hypertension		14 (20.9%)
	Coronary heart disease		1 (1.5%)
	Diabetes		10 (14.9%)
Smoking history			24 (35.8%)
Drinking history			25 (37.3%)
Disease severity			
	Admission to ICU		54 (80.6%)
	SIRS		2 (2–3)
	APACHE II score		11 (8–13)
	CTSI within 1 week of onset		4 (3–6)
	IL-6		122.0 (45.5–261.0)
	C-reactive protein (mg/L)		162.5 (82.3–317.3)
	White cell count (×10^−^⁹/L)		13.8 (5.9)
	AMY		921 (691)
	Organ failure		46 (68.7%)
	Persistent organ failure		33 (49.3%)
	Respiratory		34 (50.7%)
	Cardiovascular		0
	Renal		8 (11.9%)
	Pancreatic necrosis		26 (38.8%)
	Infected pancreatic necrosis		12 (17.9%)
	MAP		10 (14.9%)
	MSAP		22 (32.8%)
	SAP		35 (52.2%)
	Death		6 (9%)

ICU, intensive care unit; SIRS, systemic inflammatory response syndrome; APACHE II, Acute Physiology and Chronic Health Evaluation II; IL-6, interleukin-6; MAP, mild acute pancreatitis; MSAP, moderately severe acute pancreatitis; SAP, severe acute pancreatitis; CTSI: computed tomography severity index; AMY, amylase.

### Serum interleukin-6 and C-reactive protein levels in patients with differing severities of acute pancreatitis

The levels of IL-6 were significantly elevated in AP patients within 48 h of onset. Compared with MAP (median 52.34 [IQR 29.4–121.5]) and MSAP (median 108.0 [IQR 42.6–206.1]) patients, SAP patients had the highest serum IL-6 level (median 173.3 [IQR 65.7–321.3], p < 0.05). Similarly, SAP patients had the highest serum CRP level (median 296.0 [IQR 153.0–377.0] whereas MSAP patients had a moderate serum CRP level (median 110 [IQR 42.3–260.0]), and MAP patients had the lowest serum CRP level (median 81.3 [IQR 43.7–102.5], p < 0.05) (**Table 3**). Spearman correlation analysis showed that the IL-6 level was significantly positively correlated with the severity of AP (p < 0.05) and the same with that of CRP (p < 0.05). CRP had a stronger correlation with severity than IL-6 (0.513 vs. 0.327) (**Table 3**).

**Table 3:** Serum interleukin-6 (IL-6) and C-reactive protein (CRP) levels in patients with acute pancreatitis and their correlation with severity.

	IL-6 (pg/ml)	CRP (mg/L)
MAP	52.34 (29.4–121.5)	81.3 (43.7–102.5)
MSAP	108.0 (42.6–206.1)	110 (42.3–260.0)
SAP	173.3 (65.7–321.3)	296.0 (153.0–377.0)
p-value^§^	<0.05	<0.05
Spearman’s rho	0.327	0.513
p-value^*^	<0.05	<0.05

MAP, mild acute pancreatitis; MSAP, moderately severe acute pancreatitis; SAP, severe acute pancreatitis.

^§^Kruskal–Wallis H.

^*^Spearman’s rho.

### Predictive value of interleukin-6 and C-reactive protein for severe acute pancreatitis, organ failure, pancreatic necrosis, infected pancreatic necrosis, and mortality

**Figure 2:**
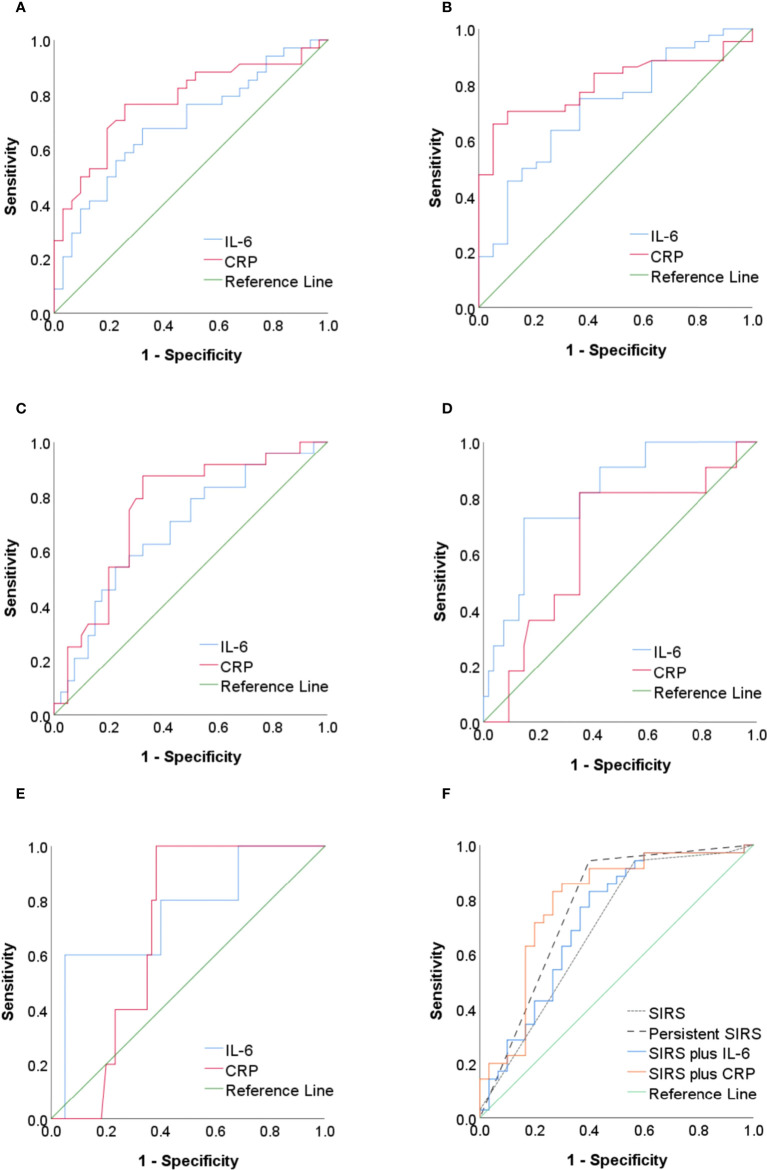
**(A–E)** The ROC curve of IL-6 and CRP as predictors of SAP, OF, PN, IPN, and mortality in AP. **(F)** The ROC curve of SIRS, persistent SIRS, IL-6 combined with SIRS, and CRP combined with SIRS as predictors of SAP. ROC, receiver operating characteristic; IL-6, interleukin-6; CRP, C-reactive protein; SAP, severe acute pancreatitis; OF, organ failure; PN, pancreatic necrosis; IPN, infected pancreatic necrosis; AP, acute pancreatitis; SIRS, systemic inflammatory response syndrome.

**Figure 2** and **Table 4** show the predictive value of IL-6 and CRP for SAP, OF, PN, IPN, and mortality. The AUC for the use of IL-6 (cutoff = 121.1 pg/ml) measured within 48 h of onset for the prediction of SAP was 0.69 (95% CI, 0.56–0.82), with a sensitivity of 67.65%, a specificity of 67.74%, a +LR of 2.10, and a −LR of 0.48. The AUCs for the use of IL-6 for the prediction of OF, PN, IPN, and mortality were 0.72 (95% CI, 0.58–0.85), 0.68 (95% CI, 0.55–0.82), 0.81 (95% CI, 0.69–0.94), and 0.75 (95% CI, 0.52–0.99), respectively. The ROC curve analysis showed that CRP was more accurate (AUC= 0.78; 95% CI, 0.66–0.89) for the prediction of SAP than IL-6 (AUC= 0.69; 95% CI, 0.56–0.82) (**Figure 2A**). The AUCs for the use of CRP for the prediction of OF (AUC 0.80; 95% CI, 0.69–0.91) and PN (AUC 0.75; 95% CI, 0.63–0.87) were also higher than those for the use of IL-6 (**Figures 2B, C**). With regard to the prediction of IPN and mortality, IL-6 was superior (AUC 0.81 and 0.75, respectively) to CRP (**Figures 2D, E**).

**Table 4:** Comparison of IL-6 and CRP for the prediction of SAP, OF, PN, IPN, and death.

	Predictive method	AUC (95% CI)	p-value	Cutoff value	Sensitivity	Specificity	+LR	−LR
SAP	IL-6	0.69 (0.56–0.82)	0.008	121.10	67.65%	67.74%	2.10	0.48
	CRP	0.78 (0.66–0.89)	0.000	142.50	76.47%	74.19%	2.96	0.32
OF	IL-6	0.72 (0.58–0.85)	0.006	54.16	75.00%	63.16%	2.04	0.40
	CRP	0.80 (0.69–0.91)	0.000	117.00	70.45%	89.47%	6.69	0.33
PN	IL-6	0.68 (0.55–0.82)	0.016	122.95	62.50%	67.50%	1.92	0.56
	CRP	0.75 (0.63–0.87)	0.001	117.00	87.50%	67.50%	2.69	0.19
IPN	IL-6	0.81 (0.69–0.94)	0.001	219.30	72.73%	85.19%	4.91	0.32
	CRP	0.65 (0.47–0.82)	0.13	170.00	81.82%	64.81%	2.33	0.28
Death	IL-6	0.75 (0.52–0.9)	0.06	122.95	80.00%	60.00%	2.00	0.33
	CRP	0.70 (0.57–0.82)	0.15	170.00	100.00%	61.67%	2.61	0.00

AUC, area under the ROC curve; CI, confidence interval; +LR, positive likelihood ratio; −LR, negative likelihood ratio; IPN, infected pancreatic necrosis; OF, organ failure; PN, pancreatic necrosis; SAP, severe acute pancreatitis; IL-6, interleukin-6; CRP, C-reactive protein.

### Systemic inflammatory response syndrome plus interleukin-6 and systemic inflammatory response syndrome plus C-reactive protein for the prediction of severe acute pancreatitis, infected pancreatic necrosis, and mortality

The AUC for the use of SIRS on the first day of admission for the prediction of SAP was 0.69 (0.56–0.82), with a sensitivity of 94.29% and a specificity of 43.33% (**Figure 2F** and **Table 5**). The AUC for the use of persistent SIRS for the prediction of SAP was 0.77 (0.65–0.89), and specificity increased to 60.00%. The combination of SIRS at admission and serum IL-6 (>121.1 pg/ml) within 48 h improved the accuracy of the prediction of SAP (AUC = 0.72), but the sensitivity (82.86%) was lower than that for persistent SIRS. The accuracy (AUC = 0.79) and specificity (73.33%) of the use of SIRS at admission combined with CRP (>142.5 mg/L) within 48 h for the prediction of SAP were higher than those of the use of SIRS plus IL-6 (**Figure 2F**). The AUC of SIRS plus IL-6 in predicting IPN and mortality was lower than that of IL-6 alone (**Supplementary Tables S1** and **S2**)

**Table 5:** Comparison of SIRS, persistent SIRS, SIRS plus IL-6, and SIRS plus CRP for the prediction of SAP.

Predictive method	AUC (95% CI)	Sensitivity	Specificity	+LR	−LR	DOR
SIRS	0.69 (0.56–0.82)	94.29%	43.33%	1.66	0.13	12.77
Persistent SIRS	0.77 (0.65–0.89)	94.29%	60.00%	2.36	0.10	23.60
SIRS plus IL-6	0.72 (0.59–0.85)	82.86%	60.00%	2.07	0.29	7.14
SIRS plus CRP	0.79 (0.67–0.91)	82.86%	73.33%	3.11	0.23	13.52

AUC, area under the ROC curve; CI, confidence interval; +LR, positive likelihood ratio; −LR, negative likelihood ratio; DOR, diagnostic odds ratio; SIRS, systemic inflammatory response syndrome; IL-6, interleukin-6; CRP, C-reactive protein.

## Discussion

This study, which was based on data from a prospectively collected AP database, found that the levels of serum IL-6 and CRP increased in proportion to the severity of AP, and both had a strong correlation with severity. Moreover, we found that compared with CRP, serum IL-6 has higher predictive accuracy for IPN and mortality but lower predictive accuracy for SAP, OF, and PN. We confirmed that the additional consideration of IL-6 improved the accuracy of the use of SIRS for the prediction of SAP at admission, although accuracy and sensitivity were lower than those obtained with the use of persistent SIRS and specificity was lower than that obtained with the use of SIRS combined with CRP.

AP is an inflammatory reaction in pancreatic tissue related to the inappropriate activation of trypsinogen to trypsin and a lack of the prompt elimination of active trypsin inside the pancreas. The activation of digestive enzymes causes pancreatic injury and results in an inflammatory response. The acute inflammatory response itself causes substantial tissue damage and may progress outside the pancreas to SIRS, multiorgan failure, or death (Whitcomb, 2006). Serum cytokine levels reflect the magnitude of the inflammatory response. IL-6 is a prototypical cytokine that has redundant and pleiotropic activity, the synthesis of which is promptly induced to aid in host defense when tissue damage or inflammation because of infections or injuries occurs (Tanaka and Kishimoto, 2014). Several studies have demonstrated an association between IL-6 and AP and found that IL-6 is a useful marker for the assessment of the severity of AP in its early stages (Inagaki et al., 1997; Jiang et al., 2004; Stimac et al., 2006; Karpavicius et al., 2016). In agreement with these previous studies, our results show that the IL-6 level is correlated with the severity of AP; the higher the IL-6 level in a patient is, the more likely the development of SAP in that patient. The present study identified a cutoff value of ≥121.10 pg/ml for IL-6, with a sensitivity of 67.65% and a specificity of 67.74%, for the prediction of SAP. This result is consistent with the results of previous studies (Schütte and Malfertheiner, 2008). Sathyanarayan et al. found that, at a cutoff value of 122 pg/ml on day 3, IL-6 has a sensitivity of 81.8% and a specificity of 77.7%, for the prediction of SAP (Sathyanarayan et al., 2007). Because the serum IL-6 concentration decreases very rapidly over time, a prospective study showed that serum IL-6 detected within 48 h of onset was the most accurate for the prediction of SAP (Jain et al., 2018). Considering the urgent need to predict the severity as soon as possible, we chose IL-6 detected within 48 h of onset for the prediction of SAP, which has more clinical relevance.

CRP has been widely adopted as a nonspecific indicator of inflammation; a number of clinical studies have reported that CRP plays an important role in the prediction of SAP (Leser et al., 1991; Jiang et al., 2004; Papachristou and Whitcomb, 2004; Sternby et al., 2017; Zheng et al., 2018). Serum CRP levels increase during the first 24 h and peak between 36 and 48 h after the onset of AP (Heath et al., 1993). Viedma et al. found that the serum CRP level was relatively high and remained high for a long time in patients with SAP. A serum CRP level of >300 mg/l can be used to predict SAP (Viedma et al., 1994). Another study indicated that a CRP level of >150 mg/l can be used to predict severe attacks of AP with a sensitivity of 90% and a specificity of 79% (Heath et al., 1993). Many studies have shown that the use of CRP to predict SAP has a sensitivity and a specificity of approximately 80% (Dervenis et al., 1999). The present study found that a CRP level greater than 142.50 mg/l could be used to distinguish between severe and mild attacks, with a sensitivity of 76.47% and a specificity of 74.19%, which was consistent with previous studies.

Over the years, several studies have been conducted to compare the use of different serum markers for the early identification of patients with AP who are at risk for severe disease. Heath et al. found that IL-6 had a sensitivity of 100% and a specificity of 71% for the prediction of SAP; CRP had a sensitivity of 90% and a specificity of 79%, indicating similar predictive value (Heath et al., 1993). Pezzilli et al. reported that CRP had a lower prognostic efficiency than IL-6 (sensitivity of 100% and specificity of 83% vs. sensitivity of 87% and specificity of 46%) (Pezzilli et al., 1999), and recently, a systematic review and meta-analysis also reported the superiority of IL-6 for the early prediction of MSAP/SAP (van den Berg et al., 2020). However, in 2012, the revised Atlanta classification recommended SIRS as one index indicating the potential for SAP and did not mention any laboratory markers that were available in clinical practice and consistently accurate for the prediction of SAP; the accuracy of IL-6 and CRP for the prediction of SAP is unclear (Banks et al., 2013). A recent study showed that IL-6 was closely related to the severity of AP, whereas CRP had low predictive accuracy for SAP (Nieminen et al., 2014). Duarte-Rojo et al. found that during the first 48 h after admission, IL-6 was more accurate than CRP (Duarte-Rojo et al., 2009). Our study revealed that IL-6 and CRP both have a strong correlation with severity, but CRP has a higher predictive value than IL-6 for the prediction of SAP (AUC 0.78 vs. AUC 0.69). Because of their low cost, ease of performance, and widespread availability, tests for CRP are generally considered to be the “gold-standard” biochemical marker for the severity of AP (Wilson et al., 1989; Papachristou and Whitcomb, 2004; Staubli et al., 2015). Our results are in accordance with this statement; a CRP level of >142.50 mg/l had a sensitivity of 76% and a specificity of 74% for the prediction of SAP. Several studies have demonstrated the predictive value of Il-6 and CRP for OF, PN, IPN, and mortality (Teerenhovi and Nordback, 1988; Ueda et al., 1997; Mándi et al., 2000; Kaya et al., 2007; Cardoso et al., 2013; Khanna et al., 2013; Karpavicius et al., 2016; Kolber et al., 2018; Vasudevan et al., 2018). A study found that IL-6 is a good marker of peripancreatic necrosis (Karpavicius et al., 2016), and previously published results showed that IPN can aggravate prognosis (Kolber et al., 2018). Our study also compared the predictive values of Il-6 and CRP for OF, PN, IPN, and mortality. The results indicated that serum IL-6 was more accurate than CRP for the prediction of IPN and mortality, but not for the prediction of OF and PN.

To mitigate the limitations inherent in the use of individual prognostic markers, some studies used combinations of multiple laboratory markers to predict the severity of AP. In 1999, Pezzilli et al. found that combining IL-6 and lipase obtained a good result with regard to the prediction of SAP (Pezzilli et al., 1999). Recently, Tian et al. reported that the combination of CRP, Procalcitonin (PCT), IL-6, and lactate dehydrogenase (LDH) is a good predictor of the severity of AP (Cardoso et al., 2013; Tian et al., 2020). SIRS is superior for the early identification of SAP; because of the low accuracy of SIRS on admission and the need to wait for 48 h for persistent SIRS, Jain et al. combined early SIRS (on admission) and IL-6 for the prediction of SAP and found that IL-6 significantly improved the predictive ability (Jain et al., 2018). Similarly, we combined early SIRS with IL-6 and CRP and compared the accuracy of those combinations with regard to the prediction of SAP; we found that both IL-6 and CRP improved the accuracy of the prediction of SAP, but SIRS at admission combined with CRP within 48 h was more accurate than SIRS plus IL-6.

This study has some limitations. First, it was a single-center study, and patients admitted to our center had relatively more severe disease (Tian et al., 2020; Yu et al., 2020). Most of the patients in our study needed intensive care, and the percentage of mild cases was lower than that observed in general hospitals. Second, hypertriglyceridemia (44.8%) was the major etiology of AP in our study, whereas previous studies showed that biliary etiology was the most common (He et al., 2017; Zhu et al., 2017; Fan et al., 2018). Severe Hypertriglyceridemic (HTG) significantly increases the severity of AP (Zhang et al., 2019), which is consistent with the observation of relatively more severe cases in our study. Finally, the small sample size is also a limitation, and a large-sample prospective study is needed for validation. Whatever, our study provides some guidance for clinicians seeking to identify patients early who are at risk for SAP, enabling them to promptly initiate therapy.

## Conclusions

In conclusion, our study found that CRP and IL-6 had diagnostic value for the severity of AP, and for predicting IPN and mortality, IL-6 had some advantages over CRP.

## Data availability statement

The raw data supporting the conclusions of this article will be made available by the authors, without undue reservation.

## Ethics statement

The construction of the AP database and the performance of this study was conducted according to the Declaration of Helsinki and was approved by the Clinical Ethics Committee of the First Affiliated Hospital of Nanchang University (approval number (2011)001). Written informed consent from the patients/participants was not required to participate in this study in accordance with the national legislation and the institutional requirements.

## Author contributions

WH designed the research. JL, ZC, LL, TL, HP and LG collected data. JL, ZC and WH analyzed the data. JL drafted the manuscript. YZ reviewed and revised the manuscript. All authors contributed to the article and approved the submitted version.

## Funding

The study design and data collection were funded by the National Natural Science Foundation of China (81860122) and Yuanhang Engineering of Jiangxi Province (1210661001).

## Acknowledgments

We want to thank the staff of the Department of Gastroenterology of the First Affiliated Hospital of Nanchang University, Nanchang, China. We are also grateful to Dr. WH for the helpful suggestions in data collection. The conference abstract of this study has been published in the *Journal of Digestive Diseases*.

## Conflict of interest

The authors declare that the research was conducted in the absence of any commercial or financial relationships that could be construed as a potential conflict of interest.

## Publisher’s note

All claims expressed in this article are solely those of the authors and do not necessarily represent those of their affiliated organizations, or those of the publisher, the editors and the reviewers. Any product that may be evaluated in this article, or claim that may be made by its manufacturer, is not guaranteed or endorsed by the publisher.

## Abbreviations

AP, acute pancreatitis; CRP, C-reactive protein; IL-6, interleukin-6; MAP, mild acute pancreatitis; MSAP, moderately severe acute pancreatitis; SAP, severe acute pancreatitis; IPN, infected pancreatic necrosis; OF, organ failure; PN, pancreatic necrosis; SIRS, systemic inflammatory response syndrome.
