# Supplementary material for: Interleukin-6 is better than C-reactive protein for the prediction of infected pancreatic necrosis and mortality in patients with acute pancreatitis
Source: Front Cell Infect Microbiol. 2022 Nov 18;12:933221. doi: 10.3389/fcimb.2022.933221 (PMC9716459; doi:10.3389/fcimb.2022.933221)
Supplement: Supplementary file 1 [file Table_1.docx]

Supplementary table1. Comparison of SIRS, persistent SIRS, SIRS plus IL-6, and SIRS plus CRP for the prediction of infected pancreatic necrosis.

| Predictive method | AUC( 95% CI) | Sensitivity | Specificity | LR+ | LR- |
| --- | --- | --- | --- | --- | --- |
| SIRS | 0.67 (0.56 - 0.78) | 0.96 | 0.38 | 1.55 | 0.11 |
| Persistent SIRS | 0.75 (0.65 - 0.85) | 0.69 | 0.72 | 2.46 | 0.43 |
| SIRS plus IL-6 | 0.77(0.68 - 0.87) | 0.91 | 0.55 | 2.02 | 0.16 |
| SIRS plus CRP | 0.75 (0.66 - 0.85) | 0.90 | 0.52 | 1.88 | 0.19 |

AUC: area under the ROC curve; CRP, C-reactive protein; DOR, Diagnostic Odds Ratio;LR+, positive likelihood ratio; LR-,negative likelihood ratio; SIRS: Systemic inflammatory response syndrome;

Supplementary table 2. Comparison of SIRS, persistent SIRS, SIRS plus IL-6, and SIRS plus CRP for the prediction of mortality.

| Predictive method | AUC( 95% CI) | Sensitivity | Specificity | LR+ | LR- |
| --- | --- | --- | --- | --- | --- |
| SIRS | 0.68 (0.56 - 0.80) | 0.96 | 0.38 | 1.55 | 0.11 |
| Persistent SIRS | 0.61 (0.49 - 0.74) | 0.69 | 0.72 | 2.46 | 0.43 |
| SIRS plus IL-6 | 0.64 (0.52 - 0.76) | 0.91 | 0.55 | 2.02 | 0.16 |
| SIRS plus CRP | 0.63 (0.51 - 0.75) | 0.90 | 0.52 | 1.88 | 0.19 |

AUC: area under the ROC curve; CRP, C-reactive protein; DOR, Diagnostic Odds Ratio;LR+, positive likelihood ratio; LR-,negative likelihood ratio; SIRS: Systemic inflammatory response syndrome;
